# The Role of Unknown Risk Factors in Myocardial Infarction

**DOI:** 10.4021/cr103e

**Published:** 2010-11-20

**Authors:** Rafighdoust Abbas Ali, Mirzaee Asadollah, Rafigdoust Amir Hossien

**Affiliations:** a Cardiology department, Imam Reza hospital, Mashhad University of Medical Sciences, Mashhad, Iran; b Cardiac surgery department, Ghaeem hospital, Mashhad University of Medical Sciences, Mashhad, Iran; c Internal Medicine, Zahedan University of Medical Sciences, Mashhad, Iran

**Keywords:** Myocardial infarction, Risk factors, Arteriosclerosis, Inflammation

## Abstract

**Background:**

Atherosclerosis of coronary arteries is the most common cause of myocardial infarction (MI), which is initiated from childhood and progresses gradually by aging. Several risk factors influence its progress, and are categorized as classic, traditional and novel factors. The role of unknown risk factors is becoming increasingly more significant recently. The aim of this study is to underscore the novel risk factors despite the importance of classic factors and consider these factors for future studies.

**Methods:**

This is a prospective study on 180 myocardial infarction cases, conducted in the cardiology ward and CCU of Imam-Reza hospital (Mashad-IRAN). A number of risk factors identified and evaluated in these patients included: hyperlipidemia, hypertension, diabetes, smoking, activity, stress, hair of external ear canal and ear lobe crease, age, and sex. Then patients without any risk factor or with one or two risk factors were distinguished.

**Results:**

The majority of our patients were old men in the age range of 60 - 69 years. Amongst all patients 42.2% were smokers, 68.3% were type A personality group, 19% were active, 81% were physically inactive, 37.2% had hairy ear canal, 35% had hypertension, 21.1% were diabetic, 14.4% had hyperlipidemia and 30% had positive family history of myocardial infarction. Of great interest was the fact that of the patients whose case was studied, many did not have any risk factor or in some cases had only one.

**Conclusions:**

In regard of increasing rate of cardiovascular diseases and myocardial infarction even amongst the young population, and because of considerable need to improve vascular risk detection, much research over the past decade has focused on identification of novel atherosclerotic risk factors, and some of these new risk factors are identified and some may be unknown. Amongst the new risk factors, inflammation has an important role, other risk factors that must be assessed are homocysteine, serum amyloid, and antibodies against Oxidized LDL. So we recommend that governments and heart associations must introduce new plans and policies in order to tackle the problem and reduce the frequency of cardiovascular disease. This requires the understanding of the conventional or classic risk factors and also the less known and new risk factors and ways which they may be prevented.

## Introduction

Atherosclerosis is the most common cause of ischemic heart diseases (IHD) that is initiated from childhood (Fatty Streak) and progresses gradually.

Several factors impact its progression, a number of which are classic or traditional factors and some unknown.

Classic or traditional risk factors include hyperlipidemia, hypertension, smoking, diabetes and metabolic syndrome.

Nevertheless, patients with ischemic heart disease are encountered without presenting any of the named risk factors in clinic. The question which arises is thus: what is the predisposing factor for atherosclerosis in these patients?

In an attempt to investigate this question we decided to evaluate patients with myocardial infarction (MI) who are without any classic risk factors.

## Materials and Methods

This is a prospective study of 180 acute MI patients conducted in the cardiology ward and CCU of Imam Reza and Ghaem hospitals. The first aim was the evaluation and identification of classic risk factors, and the second was the assessment of those patients without any known risk factor. Patients were thus divided into different age groups, and age and sex distribution of MI was evaluated.

Classic risk factors included age, hyperlipidemia, hypertension, diabetes, smoking, physical activity, familial history of MI, and finally ear canal hair and ear lobe crease.

## Results

Among the 180 MI patients, 70.6% were male and 32.1% were aged between 60 and 69 years (Table 1).

**Table 1:** Age and Sex Distribution in MI Cases

Age Group	Total N (%)	Female N (%)	Male N (%)
20 - 29	3 (1.7%)	0 (0.0%)	3 (100%)
30 - 39	7 (3.9%)	1 (14.3%)	6 (85.7%)
40 - 49	23 (12.8%)	5 (21.7%)	18 (78.3%)
50 - 59	48 (26.7%)	11 (22.9%)	37 (77.08%)
60 - 69	58 (32.1%)	20 (34.5%)	38 (65.5%)
70 - 79	34 (18.9%)	15 (44.1%)	19 (55.9%)
80 - 89	7 (3.9%)	1 (14.3%)	6 (85.9%)
Sum	180 (100%)	53 (29.4%)	127 (70.6%)

Seventy-six patients (42.2%) were smoker (Table 2) and Type A personality was seen in 123 patients (68.3%) (Table 3). Physical activity as daily exercise was seen only in 33 cases (18.3%) but poor physical activity was reported in 147 cases (81.7%) (Table 4). Ear canal hair was seen in 67 patients (37.2%) and 8 cases (4.4%) had helical fold and both were reported in 7 cases (4/2%) (Table 5).

**Table 2:** Smoking in Different Age Groups

Age Group	Less than 10 Years Smoking N (%)	10 - 20 Years Smoking N (%)	More than 20 Years Smoking N (%)
20 - 29	2 (14.3%)	0 (0.0%)	0 (0.0%)
30 - 39	1 (7.1%)	1 (7.7%)	2 (4.2%)
40 - 49	3 (21.4%)	4 (30.8%)	4 (8.3%)
50 - 59	2 (14.3%)	4 (30.8%)	12 (25%)
60 - 69	3 (21.4%)	2 (15.4%)	18 (37.5%)
70 - 79	3 (21.4%)	1 (7.7%)	11 (22.9%)
80 - 89	0 (0.0%)	1 (7.7%)	1 (2.08%)
Sum	14 (18.4%)	13 (17.1%)	48 (63.1%)

**Table 3:** Frequency of Stress in Different Age Groups

Age Group	Number of Patients With Stress	Percentage
20 - 29	3	2.4
30 - 39	6	4.9
40 - 49	19	15.4
50 - 59	34	27.6
60 - 69	37	30.08
70 - 79	20	16.3
80 - 89	4	3.3
Sum	123	68.3

**Table 4:** Physical Activity in Different Age Groups

Age Group	Number of Patients With Physical Activity	Percentage
20 - 29	0	0.0
30- 39	2	6.06
40 - 49	5	15.2
50 - 59	14	42.4
60 - 69	7	21.2
70 - 79	5	15.2
80 - 89	0	0.0

**Table 5:** Ear Canal Hair and Helical Fold in Different Age Groups

Age Group	Hairy Ear Canal N (%)	Helical Fold N (%)	Both N (%)
20 - 29	-	0 (0.0%)	-
30 - 39	2 (3%)	0 (0.0%)	1 (5.9%)
40 - 49	9 (13.4%)	0 (0.0%)	1 (5.9%)
50 - 59	19 (28.3%)	4 (50%)	1 (5.9%)
60 - 69	19 (28.3%)	3 (37.5%)	7 (41.2%)
70 - 79	15 (22.4%)	1 (12.5%)	6 (35.3%)
80 - 89	3 (4.5%)	0 (0.0%)	1 (5.9%)
Sum	67 (37.2%)	8 (4.4%)	17 (9.4%)

Thirty-eight patients (21.1%) were diabetic and hypertension was seen in 35% (Table 6, 7).

**Table 6:** Frequency of DM in Different Age Groups

Age Group	Total Diabetic Patients N (%)	DM for 0 - 10 Years N (%)	DM for 10 - 20 Years N (%)	DM of More than 20 Years N (%)
20-29	0 (0.0%)	0 (0.0%)	0 (0.0%)	0 (0.0%)
30-39	1 (2.6%)	1 (3.4%)	0 (0.0%)	0 (0.0%)
40-49	2 (5.3%)	2 (6.9%)	0 (0.0%)	0 (0.0%)
50-59	11 (28.9%)	10 (34.5%)	1 (12.5%)	0 (0.0%)
60-69	19 (50.0%)	12 (41.4%)	6 (75%)	1 (100%)
70-79	5 (13.1%)	4 (3.8%)	1 (12.5%)	0 (0.0%)
80-89	0 (0.0)	0 (0.0%)	0 (0.0%)	0 (0.0%)
Sum	38 (31.1%)	29 (76.3%)	8 (21.5%)	1 (2.6%)

**Table 7:** Frequency of Htn in Different Age Groups

Age Group	Number of Hypertensive Cases	Percentage
20 - 29	0	0.0
30 - 39	0	0.0
40 - 49	4	6.3
50 - 59	13	20.6
60 - 69	26	41.3
70 - 79	17	26.9
80 - 89	3	4.8
Sum	63	35

Lipid profile in 20 - 39 year age group showed some cases with cholesterol level higher than 200 mg/dl, and 17% of patients in 40 - 89 year age group had cholesterol level higher than 200 mg/dl (Table 8).

**Table 8:** Cholesterol Level in Different Age Groups

Age Group	Chol ≥ 200mg/dl (%)	Chol < 200mg/dl (%)
20 - 29	0	100
30 - 39	0	100
40 - 49	17	83
50 - 59	17	83
60 - 69	16	84
70 - 79	15	85
80 - 89	0	100

LDL level in all patients with the age of 20 - 39 years was lower than 160 mg/dl but 35% of 40 - 80 year age group had higher than 160 mg/dl (Table 9).

**Table 9:** LDL Level in Different Age Groups

Age Group	LDL ≥ 160mg/dl (%)	LDL < 160mg/dl (%)
20 - 29	0	100
30 - 39	0	100
40 - 49	35	65
50 - 59	29	71
60 - 69	20	80
70 - 79	27	73
80 - 89	31	69

Finally, positive family history was seen in 55 patients (30.5%) (Table 10).

**Table 10:** Family History of IHD in Different Age Groups

Age Group	Frequency of Positive FH for IHD	Percentage
20 - 29	2	3.6
30 - 39	5	9.09
40 - 49	9	16.4
50 - 59	16	29.09
60 - 69	16	29.09
70 - 79	7	12.7
80 - 89	0	-
Sum	55	30.5

Combination of risk factors including hyperlipidemia, hypertension, diabetes and smoking were not reported in patients in the age range of 20 - 39 years, and these risk factors were seen on average in 25% of 30 - 89 year age group (Table 11).

**Table 11:** Frequency of Patients Without Combination Risk Factors for IHD (Htn, HPL, DM and Smoking) in Different Age Groups

Age Group	Patients Without Any Risk Factor	Percentage
20 - 29	3	100
30 - 39	7	14
40 - 49	23	17
50 - 59	48	29
60 - 69	58	18
70 - 79	34	35
80 - 89	7	-

Lipid profile showed normal LDL and total Cholesterol in 20 - 39 year age group, and normal cholesterol and LDL level were reported respectively in 83% and 70% among those patients who aged 40 - 89 years (Table 8, 9).

## Discussion

Cardiovascular disease is the most common cause of mortality in developed countries and almost one million deaths are reported annually in the US because of ischemic heart diseases (IHD) [1]. In this study we evaluated atherosclerosis risk factors in two parts. The first part consisted of epidemiological study of classic or traditional risk factors.

Nevertheless, one must mention based on long term experience that myocardial infarction may also occur in people without any known risk factor. Hence we decided to evaluate the frequency of MI in patients without any risk factor as the second part of our study [2-5].

A prospective study of healthy American ladies reported that 77% of ischemic heart diseases have occurred in patient with LDL lower than 160mg/dl and 46% have occurred in those with LDL lower than 130mg/dl [1, 6].

Another study showed that, although serum lipids have a great role in ischemic heart disease, half of patients with acute MI had normal lipid profile; and another study showed that 20% of ischemic heart diseases happened in the absence of any classic risk factor [7, 8].

Another study on known cases of ischemic heart disease did not show combination of risk factors including hyperlipidemia, hypertension, diabetes and smoking. In 15% of men, 19% of women and more than half of all patients had only one classic risk factor [9-11].

Finally, another study reported only one risk factor in 85-95% of ischemic heart disease cases [1-13].

Similarly, our study did not show combination of risk factors including hyperlipidemia, hypertension, diabetes and smoking in 20 - 29 year age group with MI, although it seems there should be a great risk factor for MI in young people.

This study showed that cholesterol was less that 200 mg/dl in all cases in 20 - 39 year age group and only 17% of patients in 40 - 89 year age group had cholesterol level higher than 200 mg/dl.

LDL level was also less than 160mg/dl in all cases in 20 - 39 year age group and only 30% of those in 40 - 89 year age group had LDL level higher than 160mg/dl. In 30 - 89 year age group, 25% of those were risk factor free. So it seems logical to detect other unknown risk factors for ischemic heart disease.

Besides genetic factors and coagulative disorders, inflammatory response becomes a new risk factor for ischemic heart disease. Inflammation without any origin or due to infection, air pollution and many other causes may promote atherosclerosis and this is of need of further studies yet [6, 12-15].

Other new risk factors include lipoprotein, homocysteine and fibrinolytic markers [13]. Fortunately, inflammatory markers are measurable and main markers including hsCRP, IL-6, P-selective sVCAM, CD40, and leukocyte activator agents such as Myeloperoxidase (MPO) could be measured exactly in laboratories [16-19].

### Conclusions

In regard of increasing rate of cardiovascular diseases and myocardial infarction even amongst the young population, and because of considerable need to improve vascular risk detection, much research over the past decade has focused on identification of novel atherosclerotic risk factors, and some of these new risk factors are identified and some maybe unknown. Amongst the new risk factors, inflammation has been recently identified which maybe the result of infection, air pollution, or stress and the like, other risk factors that must be assessed are homocysteine, serum amyloid, and antibodies against Oxidized LDL. So we recommend that governments and heart associations must introduce new plans and policies in order to tackle the problem and reduce the frequency of cardiovascular disease. This requires the understanding of the conventional or classic risk factors and also the less known and new risk factors and ways which they may be prevented.
